# Ectopic Germinal Centers and IgG4-Producing Plasmacytes Observed in Synovia of HLA-B27+ Ankylosing Spondylitis Patients with Advanced Hip Involvement

**DOI:** 10.1155/2015/316421

**Published:** 2015-04-12

**Authors:** Xiugao Feng, Xiangjin Xu, Yue Wang, Zhiyong Zheng, Guiying Lin

**Affiliations:** ^1^Department of Rheumatology, Fuzhou General Hospital of Nanjing Command, PLA, Fuzhou 350025, China; ^2^Department of Pathology, Fuzhou General Hospital of Nanjing Command, PLA, Fuzhou 350025, China

## Abstract

*Introduction*. Ectopic lymphoid neogenesis and the presence of IgG4-positive plasmacytes have been confirmed in chronic inflammatory sclerosing diseases. This study aims to investigate hip synovial tissues of ankylosing spondylitis (AS) patients for IgG4-positive plasma cells and ectopic lymphoid tissues with germinal centers (GCs). *Methods*. Synovial samples were collected from 7 AS patients who received total hip replacement and were evaluated using immunohistochemistry for the presence of CD20+ B-cells, CD3+ T-cells, CD21+ follicular dendritic cells (FDC), and CD38+ plasma cells. Furthermore, immunoglobulin G (IgG and IgG4), IgA, IgM, and complement components C3d and C4d in synovia were evaluated. Both synovial CD21+ FDCs and IgG4-producing plasmacytes were analyzed. *Results*. All seven patients had severe fibrosis. Massive infiltrations of lymphocytes were found in 5 out of 7 patients' synovia. Ectopic lymphoid tissues with CD21+ FDC networks and IgG4-positive plasma cells were observed coincidentally in two patients' synovia. *Conclusion*. The pathophysiological mechanism of AS patients' hip damage might be related to the coincidental presence of ectopic lymphoid tissue with FDCs network and IgG4-positive plasma cells identified here for the first time in AS patients' inflamed synovial tissue.

## 1. Introduction

Hip involvement is strongly associated with functional impairment in AS patients [1]. Radiological changes of the hips occur in about 30% of AS patients [2] often resulting in functional impairment. In cases with end-stage hip disease [3], hip replacement is commonly required. In affected joints, inflamed synovia might evolve from early inflammatory infiltrations to lymphoid neogenesis and eventual fibrosis or fibrosclerosis. In 1982, Revell and Mayston [4] observed considerable lymphoid cell infiltration, lymphoid follicle formation, IgG-, IgA-, and IgM-containing plasma cells, and a varying extent of fibrosis in joints from AS patients. Appel et al. also found lymphocytic infiltrates in the sacroiliac joint [5]. However, the pathogenesis of hip involvement in AS is still unclear and few have focused on it in current reports.

Ectopic lymphoid tissue, also known as tertiary lymphoid tissue, has been observed recently in chronically inflamed tissue [6, 7] where ectopic germinal centers (GCs) have been identified and are characterized by follicular dendritic cell (FDC) networks. Current limited studies reported ectopic lymphoid tissue to be related to the increased severity of disease [7, 8] and the production of autoantibodies [9]. Ectopic lymphoid tissue with GCs and its pathophysiology remain to be investigated extensively in inflammatory diseases, especially in those with lymphoid follicle formation in inflamed tissues.

On the other hand, the presence of IgG4 in some chronic inflammatory sclerosing diseases has been confirmed. The designation “IgG4-Related Sclerosing Diseases, IRSDs” has been proposed [10, 11]. ISRDs refer to a collection of various conditions and clinically respond well to steroid therapy, reportedly. Prominent pathological features include massive infiltrations of IgG4-producing plasma cells, lymphoid follicle formation, and fibrosclerosis in single or multiple organs. The IgG4-producing plasma cells can be detected in other autoimmune inflammatory diseases as well [12]. AS is currently considered an inflammatory joint disease; however, lymphoid follicles, IgG-, IgA-, and IgM-containing plasma cells have been found in inflamed peripheral joints [4]. It is unclear whether inflamed joints in AS have pathological changes similar to findings in autoimmune inflammatory diseases. This study aims to investigate the synovial tissue of AS patients' hips with end-stage disease, using immunohistochemistry to detect the possible presence of ectopic lymphoid tissue with GCs and IgG4-positive plasma cells.

## 2. Methods 

### 2.1. Patient Selection

Seven AS patients undergoing total hip replacement surgery in 2010-2011 for severely damaged hip joints were selected for study inclusion. Patients with ischemic femoral head necrosis or osteoarthritis were excluded. All patients met the modified New York criteria for ankylosing spondylitis [13] and had no evidence of autoimmune rheumatic disease nor IgG4-related diseases. No patients had a history of drinking alcohol. The Ethics Committee of Fuzhou General Hospital approved the study. Informed consent was obtained from patients. Clinical data including gender, age, disease duration, erythrocyte sedimentation rate (ESR), C-reactive protein (CRP) level, and radiographic data were collected. Radiographic evaluation of hip joints was performed and joint spaces were measured. Synovial tissue samples were obtained and sent for pathological examination following total hip replacement.

### 2.2. Synovial Tissue Handling

After surgery, synovial tissue was obtained from joints by longitudinally cutting the synovium along the surface of the femoral head and fixed in 40% formaldehyde for 24 hours. Then, the synovial tissue was embedded in paraffin and stored at room temperature. Prior to continued preparation and evaluation, 3 *μ*m sample sections were deparaffinized with xylene and then rehydrated with 100%, 95%, and 80% ethanol.

### 2.3. Synovial Tissue Preparation and Evaluation

*HE Staining*.  Staining with hematoxylin and eosin was performed before evaluation for synovial histopathological changes.

*Evaluation*.  Synovial tissue evaluation was made by an experienced observer at X200 magnification against 5 consecutive fields.

### 2.4. Immunohistochemical Detection

3 *μ*m sections of sample were deparaffinized with xylene and then rehydrated with 100%, 95%, and 80% ethanol. Slides were treated with citrate sodium at 95°C for 10 min and then incubated with 3% H_2_O_2_. Primary antibodies were applied to the slides overnight at 4°C, followed by incubation with horseradish peroxidase-labeled secondary antibodies for 1 h at room temperature with repeated washes in between.

### 2.5. Immunohistochemical Detection of CD+ Cells

DAB was applied for development. Primary antibodies included rabbit anti-human CD3 polyclonal antibody, mouse anti-human CD20 monoclonal antibody, mouse anti-human CD21 monoclonal antibody, and mouse anti-human CD38 monoclonal antibody. All antibodies were purchased from LSBio (Maixing Company, China). Five random fields at 400x magnification were photographed for each slide. Positive cells were counted and averaged (Table 2).

### 2.6. Immunohistochemical Assay for Plasma Cells Secreting IgG, IgG4, IgA, and IgM

AEC was applied for development. Primary antibodies included rabbit anti-human IgA polyclonal antibody (DAKO), rabbit anti-human IgM polyclonal antibody (DAKO), rabbit anti-human IgG polyclonal antibody (DAKO), mouse anti-human IgG4 monoclonal antibody (Invitrogen), rabbit anti-human C1q polyclonal antibody (DAKO), rabbit anti-human C3d polyclonal antibody (Abcam), and rabbit anti-human C4d polyclonal antibody (Biomedica). Horseradish peroxidase-labelled secondary antibodies were purchased from LSBio (Maixing Company, China). For plasma cells expressing IgG and IgG4 assessment, five random fields were checked and photographed for each slide, whereby positive cells were counted and averaged. Primary isotopes were used as negative controls.

## 3. Results

### 3.1. Clinical Data

**Figure 1:**
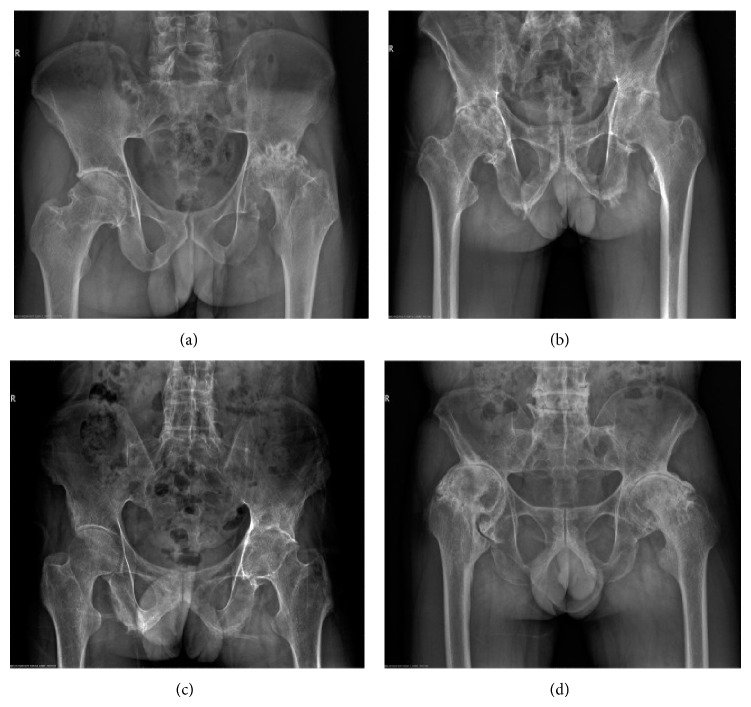
Radiographic findings in AS patients with advanced hip joint disease. (a) Case 3; (b) case 5; (c) case 6; and (d) case 7.

Preoperative characteristics of patients are listed in Table 1. The mean age and disease duration of patients were 37.1 ± 9.0 years (range: 24–52 years) and 13.7 ± 12.4 years (range: 2–40 years), respectively. HLA-B27 was positive in all patients. RF and ANA were negative. No patient had a history of iritis. Five patients had elevated ESR (5/7, 71.4%). Six patients had increased CRP (6/7, 85.7%). Radiographic evaluation of hips showed narrowed joint spaces ranging from 0 to 7 mm, including 3 of 7 patients with complete disappearance of the joint space (Figure 1). Serum levels of IgG and IgG4 were not measured.

### 3.2. Histological Findings and Expression of CD+ Immune Cells in Inflamed Synovia

**Figure 2:**
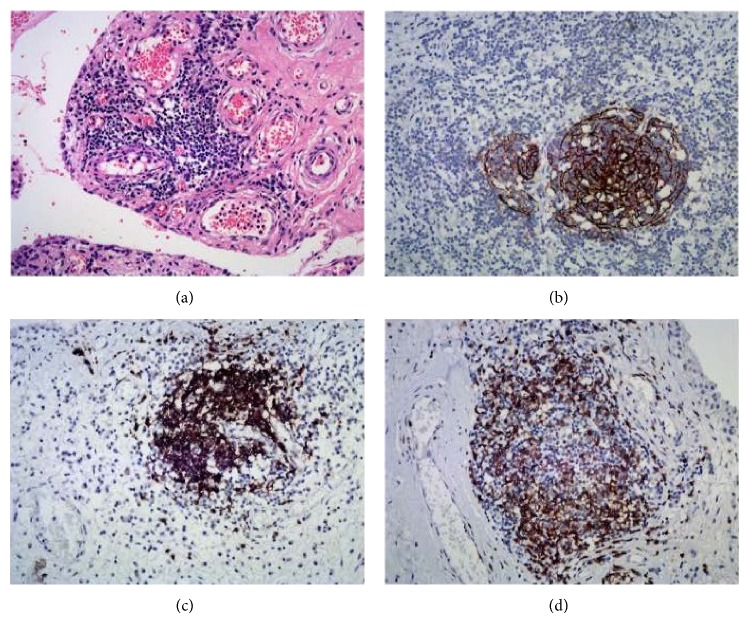
Histologic findings and expression of CD molecules in case 5. (a) Staining with hematoxylin and eosin [H&E]. (b) CD21-positive FDC networks (DAB staining, 200x); (c) numerous CD20 positive B-cells in lymphoid neogenesis (DAB staining, 200x); and (d) Numerous CD3 positive T-cells in lymphoid neogenesis (DAB staining, 200x).

Obvious fibrosis was present in all 7 cases, as was a varying infiltration of inflammatory cells. Synovial lining (intimal cell) hyperplasia was observed in 5 cases but was nearly unobservable in 2 cases with complete fibrosis. Polymorphonuclear cells were present in the field. Marked fibrin deposits were seen in all cases. New blood vessels were observed. No vasculitis nor vessel wall necrosis was observed though thickened vascular wall was present. Massive presence of inflammatory cells as well as moderate to severe synovial tissue fibrosis was observed in 5 patients; two had lymphoid neogenesis (Figure 2(a)); two patients showed complete fibrosis with few inflammatory cells infiltrating.

**Figure 3:**
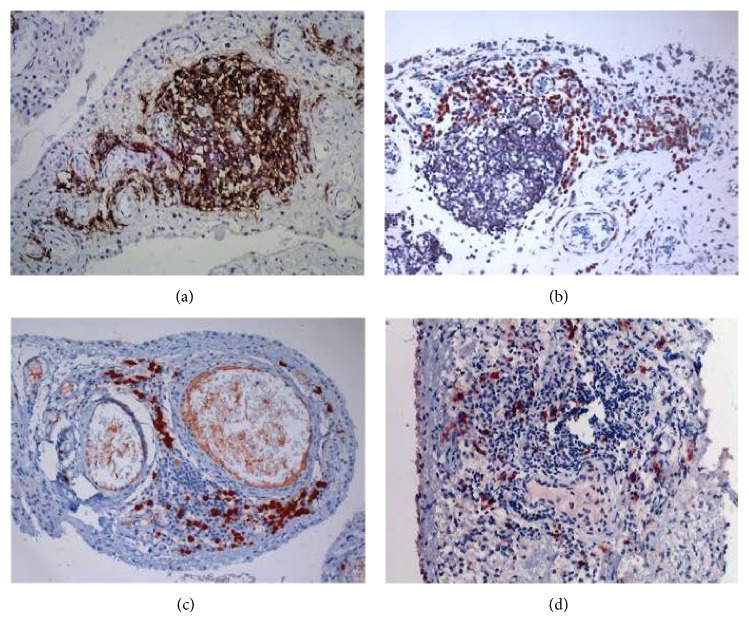
Plasma cells in synovial tissue. (a) CD38 positive plasma cells near lymphocyte follicles in case 5 (DAB staining, 200x); (b) massive IgG-positive plasma cell presence in case 5 (AEC staining, 200x); (c) numerous IgG4-positive plasma cell in case 5 (AEC staining, 200x); and (d) IgG4-positive plasma cells in case 6 (AEC staining, 200x).

Using anti-CD molecule antibodies, lymphoid-like structures were observed in two cases, consisting of a typical CD21+ FDCs network (Figure 2(b)), numerous CD20+ cells (Figure 2(c)) and CD3+ T-cells (Figure 2(d)). CD3+ T-cells were also found in the area of lymphocyte aggregation. CD38+ plasma cells were found primarily nearby lymphoid-like structures and in the surrounding small vessels (Figure 3(a)). No CD21+ dendritic cells were observed in cases without ectopic lymphoid tissue.

### 3.3. Immunoglobulin-Producing Plasma Cells in Synovial Tissue

The evaluation of immunoglobulin-producing plasma cells revealed that IgG-positive plasma cells (Figure 3(b)) were more predominant compared with IgM- and IgA-positive plasma cells. IgG4-producing plasma cells were observed only in patients with ectopic lymphoid tissues (Figures 3(c) and 3(d)). One patient had IgG4-positive cells averaging 99/HPF (Figure 3(c)), with a percentage of IgG4^+^/IgG^+^ of 69.1%. Another case had fewer IgG4-expressing plasma cells with only 5.9% in IgG-positive cells (Figure 3(d)). C1q, C3d, and C4d positive cells were barely observed except a few in endothelial cells.

## 4. Discussion

A previous study of the synovia of 14 patients who underwent synovectomy or hip replacement surgery showed that lymphocyte aggregation was present in all patients' synovia and lymphocyte follicle formation occurred in over half the samples [4]. In comparison, our patients undergoing total hip replacement presented with more extensive fibrosis and less lymphocyte follicle formation.

In our study, both ectopic lymphoid tissue with germinal centers and IgG4-producing plasma cells (Figures 3(c) and 3(d)) were identified among 2 cases. Ectopic germinal centers in these cases demonstrate a structurally distinct feature of CD21+ FDC networks (Figure 2(b)) as described by Humby et al. [14]. In several reports, either ectopic lymphoid tissue or ectopic germinal centers have been related to disease severity in several autoimmune diseases [15, 16]. The ectopic lymphoid structures with FDC networks have also been reported to support the ongoing production of class-switched autoantibodies [14]. In AS, links like these remain to be elucidated. The coincidental presence of ectopic GCs and IgG4-producing plasma cells in this study might suggest a link between them.

Regarding the IgG4-producing plasma cells, their proportions to IgG+ cells were 69.1% and 5.9% for the two cases. These observations are pathologically in accordance with the feature of IRSDs mentioned by both Kamisawa and Okamoto and Cheuk and Chan [10, 11] and hint at the clinical rationale of corticosteroid therapy. In IRSDs [10–12], the pathological role of IgG4-expressing plasmacytes is not clear at present, but the frequent coincidence with fibrosis might suggest an association in pathological processes. Above all, findings of ectopic lymphoid tissue with germinal centers and IgG4-producing plasma cells might account for the pathophysiology of severe hip joint damage in AS patients. Considering that IgG4 positivity was observed in only 2 of the 7 AS patients' synovia, future studies should be planned to carefully examine the entire joint synovia of a larger sample to determine if these findings are incidental or of pathomechanistic significance.

## 5. Conclusion

The pathogenesis of severe hip joint damage in AS patients is not yet clear. Our current study is the first to identify ectopic lymphoid tissue with FDCs networks and IgG4-positive plasma cells in the inflamed synovial tissue of AS patients with end-stage hip joint disease. This discovery may be a clue to better understand the mechanisms of the structural destruction of hip joints in AS, particularly among the HLA-B27+ patient population.

## Figures and Tables

**Table 1:** Clinical data.

Data	Age (years)	Disease duration (year)	ESR (mm/1 h)	CRP (mg/L)	BASRI	Joint space (mm)
1	24	8	22	9.8	4	0
2	35	12	34	24.9	4	0
3	32	2	25	21.2	4	0
4	41	10	17	11.4	4	0–2
5	43	16	38	50.9	4	0.5–5
6	33	8	37	49.4	4	1–7
7	52	40	11	4.79	4	0–1.5

**Table 2:** Histochemical assessment.

Data	Fibrosis	CD3	CD20	CD21	CD38	IgG	IgG4	IgM	IgA
1	2	49.2	44	0	4.2	1.3	0	0	1
2	2	81.8	46.4	0	10	6	0	1	1.3
3	3	5.2	6.2	0	9.6	6	0	1	1
4	2	51.4	25.8	0	2	0	0	0	0
5	2	343	510	79	208	143	99	60	15
6	2	503	510	224	217	187	11	10	12
7	3	3	2.7	0	0	0	0	0	0

## Abbreviations

Ig: Immunoglobulin
AS: Ankylosing spondylitis
CD: Cluster of differentiation
RF: Rheumatoid factor
ANA: Antinuclear antibody
IRSDs: IgG4-related sclerosing diseases
GC: Germinal center
FDC: Follicular dendritic cell
CD3: T-cell marker
CD20: B-cell marker
CD21: FDC marker
CD38: Plasma cell marker.
